# Oridonin inhibits pancreatic cancer cell migration and epithelial-mesenchymal transition by suppressing Wnt/β-catenin signaling pathway

**DOI:** 10.1186/s12935-016-0336-z

**Published:** 2016-07-22

**Authors:** Qian-Qian Liu, Ke Chen, Qiao Ye, Xiao-Hua Jiang, Yun-Wei Sun

**Affiliations:** Department of Gastroenterology, Ruijin Hospital, Shanghai Jiaotong University School of Medicine, Shanghai, 200025 China; School of Biomedical Sciences, Faculty of Medicine, The Chinese University of Hong Kong, Hong Kong SAR, China

**Keywords:** Oridonin, Pancreatic cancer, Metastasis, Epithelial mesenchymal transition, Wnt/β-catenin pathway

## Abstract

**Background:**

Oridonin (ORI) can inhibit proliferation and migration in various types of cancer cell lines. However, the exact mechanism remains unclear. We investigated the migration inhibitory effect of ORI on human pancreatic cancer SW1990 cells and dissected the possible molecular mechanism(s).

**Methods:**

CCK-8 assay was used to observe the cell viability. Wound healing assay, transwell assay and spontaneous metastasis model were used to observe the migration activities. Real-time PCR, immunofluorescence, western blot analysis and immunohistochemistry methods were used to observe the expression of genes or proteins.

**Results:**

ORI inhibited the migration of SW1990 cells. Real-time PCR and immuno-fluorescence analyses of epithelial-to-mesenchymal transition (EMT) markers were compared between control group and ORI group. The expression of mesenchymal molecular markers, such as vimentin, snail and slug decreased. The expression of epithelial-related marker E-cadherin increased. Wnt/β-catenin signalling was inhibited by ORI using luciferase reporter assay. ORI can decrease the β-catenin protein level not only in the nucleus, but also in the cytoplasm and the whole cell after the treatment with ORI and glycogen synthase kinase 3β (GSK3β) was increased in the ORI-treated group. CHIR could attenuate the effects of ORI in SW1990 cells. We established a mice model by injecting 1 × 10^6^ SW1990 cells into nude mice intraperitoneally to test whether ORI affects tumour metastasis. Metastatic formation was inhibited by ORI (5 and 10 mg/kg) compared with the control group. Tumour sections stained with anti-E-cadherin, anti-vimentin and anti-β-catenin antibodies revealed that ORI inhibited EMT, as well as the Wnt/β-catenin pathway in vivo.

**Conclusions:**

ORI can inhibit pancreatic cancer cell SW1990 migration and EMT by down-regulating Wnt/β-catenin signal transduction in vitro and in vivo. Therefore, it can be potentially and effectively used in the clinical management of pancreatic cancer.

## Background

Pancreatic cancer, which is one of the most common cancers of the digestive system, is a leading cause of morbidity and mortality in both developed and developing countries. Pancreatic cancer is characterised by a covert anatomical structure, aggressive biology, resistance to conventional therapeutic agents, and no early detectable biomarkers were observed [1]. Consequently, pancreatic cancer presents a great challenge in oncology, with a 5-year survival rate of only 5 % [2]. This is true for metastatic diseases, wherein the average life expectancy is just three to 6 months. Treatment options for pancreatic cancers are limited. Less than 20 % of patients with pancreatic cancer are subjected to surgical removal, whereas other patients are typically treated with chemotherapy or chemotherapy with radiation. Pancreatic cancer is one of the few cancers in which survival has not improved substantially in 40 years. Therefore, searching for new strategies to prevent and treat pancreatic cancer is essential.

The epithelial-to-mesenchymal transition (EMT) is among the most critical processes that occur during the progression of tumour metastasis. EMT-induced adenocarcinoma cells can acquire malignant features, such as invasion, metastatic capabilities and chemo-resistance [3, 4]. In human pancreatic tumour samples, fibronectin and vimentin are increased in high-grade tumours, with a corresponding decrease in E-cadherin expression. These patients have worse prognoses than those who demonstrate less evidence of EMT. Primary tumours with mesenchymal features (75 % of the total number of tumours) developed metastatic lesions to the liver and lungs [5]. Numerous signalling pathways that are involved in the regulation of EMT, such as transforming growth factor-beta, notch and Wnt signalling pathways, are highly activated in metastatic pancreatic cancers and appear to be associated with prognosis [6–8].

Oridonin, a tetracycline diterpenoid compound extracted from the traditional Chinese medicine *Rabdosia rubescens*, has anti-inflammatory, antibacterial and antitumour effects [9]. ORI inhibits the development of various types of cancers and is an effective and low-toxic antitumour medicine [10–12]. However, mechanisms underlying the antitumour activities of ORI, and whether or not it can suppress the migration of pancreatic cancer remain largely unknown.

We investigated the effects of ORI on pancreatic cancer cells. ORI inhibits pancreatic cancer cell migration and EMT in vitro and in vivo by suppressing the Wnt/β-catenin signalling pathway.

## Methods

### Chemicals and reagents

ORI was purchased from the Yuanye Biological Institutions (Shanghai, China). Its purity was measured by HPLC and determined to be 99 %. ORI was dissolved in dimethyl sulfoxide (DMSO) to make a stock solution (10 mM) and stored at −20 °C. The DMSO concentration was kept below 0.1 % in all experiments and did not exert any detectable effect on cell growth or cell death. Fetal bovine serum (FBS), 0.25 % trypsin containing EDTA and high glucose DMEM were obtained from Gibco (USA). Cell Counting Kit-8 (CCK-8) was purchased from DoJinDo (Japan). RNA extraction kit was purchased from Life Technologies Co. CHIR99021, a GSK3β inhibitor (R&D), was added into medium at a concentration of 2 μM 1 h before administration of ORI.

### Cell culture

Pancreatic cancer cell lines (Aspc1, Bxpc3, Panc1, SW1990) was obtained from the American Type Culture Collection (ATCC; Manassas, VA, USA). The cells were cultured in DMEM supplemented with 10 % FBS, 100U/ml penicillin, and 100 μg/ml streptomycin at 37 °C in a humidified atmosphere of 5 % CO_2_, and subcultured when confluency reached 70–80 % by 0.25 % trypsin.

### Cytotoxicity assay

The cytotoxic effect of ORI on pancreatic cancer cells was determined by using CCK-8 kit. Briefly, the logarithmic phase cells were plated in 96-well culture plates (5 × 10^3^ cells per well). After 24 h of incubation, cells were treated with vehicle alone (0.1 % DMSO) or various concentrations (7, 15, 31, 62, 125, 250 μM) of ORI, followed by a 48 h-culture. Each group had 6 wells, and CCK-8 (100 μl) was added to each well 1 h before the end of incubation. The absorbance at 450 nm was read using Bio-Tek, ELX800. Experiments were repeated three times. The cytotoxic effect was expressed as a relative percentage of cell death calculated as follows: Cell death (%) = 100 −(dosing absorbance − blank absorbance)/(control absorbance − blank absorbance) × 100.

### Cell migration assay

A wound scratch assay was performed in order to study the migration of SW1990 cells. A 6-well plate was seeded with cells at 60 % confluence. After overnight incubation, a scratch was made at the centre of each well using a 200 μl pipette tip. Cells were further incubated with 0, 7 or 15 μM ORI. Three parallel lines were drawn at the border of the wound, and the distance between the lines were measured 24 h post-scratching. The microscopic magnification (100×) of this area was photographed. The wound healing effect was determined by measuring the percentage of the migrating area compared with the area of the initial wound. Each experiment was conducted in triplicate.

### Transwell migration assay

SW1990 cells were subjected to a transwell migration assay. 1 × 10^5^ cells were added to the upper chamber, and the lower chamber was filled with 7 or 15 μM ORI of culture medium containing no FBS. After incubation for 24 h, cells in the upper chamber were carefully removed with a cotton swab, and cells that passed through the membrane to the lower chamber surface were fixed and stained with 0.5 % crystal violet. Cells from each well were counted in 3 random high-power fields under a microscope (100×). Each experiment was repeated in triplicate.

### Real-time PCR

Total cellular RNA was extracted using TRIzol (Invitrogen) reagent. The reverse transcription was performed with 2 μg of total RNA, using an oligo (dT) primer and other reagents, and cDNA was synthesized using the Revert Aid First-Strand cDNA Synthesis kit (Thermo Fisher Scientific, Waltham, MA, USA) according to the manufacturer’s protocol. Real-time PCR was carried out using the miScript SYBR Green PCR kit (Qiagen, Venlo, Netherlands). The cycle conditions for the PCR reaction were as follows: 95 °C for 15 min followed by 40 cycles of 60 °C for 20 s, and 72 °C for 40 s. The expression of each RNA was normalized to that of the internal control glyceraldehyde 3-phosphate dehydrogenase (GAPDH). The specific primer pairs used in this PCR reaction were: E-cadherin (NM_004360.3), forward: 5′-ACAGCCCCGCCTTATGATTCTC-3′, reverse: 5′-AAGCGATTGCCCCATTCG-TT-3′; vimentin (NM_003380.3), forward: 5′-AGATGGCCCTTGACATTGAG-3′, reverse: 5′-CCAGAGGGAGTGAATCCAGA-3′; snail (NM_005985.3), forward: 5′-AATCGGAAGCCTAACTACAGCG-3′, reverse: 5′-GTCCCAGATGAGCATT-GGCA-3′; slug (NM_003068.4), forward: 5′-GTCCGCTCTGCCGCACCTGA-3′, reverse:5′-GCTTGAGGGTCTGAATCTTGCT-3′; GAPDH (BC026907.1), forward: 5′-CTGCACCACCAACTGCTTAG-3′, reverse: 5′-GTCTTCTGGGTGGCAGTGAT-3′. The changes of expression were calculated using the (2 ^[−ΔΔCT]^) method. Each plate was run in triplicate.

### Immunofluorescence

After treated with or without ORI, SW1990 grown on 6-well plate were fixed in 4 % (w/v) paraformaldehyde and permeabilized with 0.1 % Triton-X 100 in PBS. E-cadherin (CST, 1:200), vimentin (CST, 1:200) and slug (CST, 1:200) were visualized by sequential incubation with indicated antibody overnight at 4 °C. Pictures were acquired by using a fluorescence microscope (Olympus).

### Western blot

40 μg proteins were separated by 10 % sodium dodecyl sulfate–polyacrylamide gel (SDS-PAGE), and blotted onto polyvinylidene difluoride (PVDF) membranes. The membranes were incubated with the primary antibodies against Histone H1 (Millipore, 1:1000), Non-phospho (Active) β-catenin (CST, 1:1000), β-catenin (CST, 1:1000), GSK-3β(CST, 1:1000), GAPDH (KangChen Bio-tech, 1:1000) at 4 °C overnight separately, and further incubated with horseradish peroxidase (HRP)-conjugated secondary antibody (1:10,000; keyGEN-BIO) for 1 h. Bands were visualized by Western Blotting Reagents (EMD Millipore, Billerica, MA, USA).

### Luciferase reporter assay

Sub-confluent SW1990 cells were seeded in 24-well plate and transfected with 0.5 μg β-catenin/Tcf4 luciferase reporter (pTop-luc) per well with Lipofectamine (Invitrogen). After incubating for 12 h, cells were treated with 0.1 % DMSO or 15 μM ORI. 24 h later, cells were lysed and subjected to luciferase assays using luciferase assay kit (Promega). Each assay was done in triplicate.

### Spontaneous metastasis model and Hematoxylin & Eosin (H&E) staining

SW1990 cells (1 × 10^6^) in 50 μl PBS were injected into the nude mice intraperitoneally. Based on the different treatments, mice were segregated into 3 groups (each group has 5 mice). ORI (5 mg/kg and 10 mg/kg) was injected intraperitoneally every day for 10 days starting from the second day of SW1990 injection. Lungs were dissected and fixed in 10 % formaldehyde after 1 month. Paraffin-embedded lungs were cut for H & E staining.

### Immunohistochemistry (IHC)

Immunohistochemistry analysis was performed on 5-mm sections of formalin-fixed paraffin-embedded lungs derived from mice. Serial sections were cut from each tissue, and further stained to evaluate the expression of EMT markers (E-cadherin and vimentin) and β-catenin (1:200).

### Statistical analysis

Quantitative results were expressed as mean ± standard deviation (SD). The statistical analysis was performed by using two-tailed unpaired *t* test (between two groups) or one-way analysis of variance (ANOVA) (among three or more groups) under the computer software SAS 9.2 (SAS Institute Inc., Cary, NC, USA). A two-tailed P value <0.05 was considered to be statistically significant.

## Results

### ORI inhibits cell migration in SW1990 pancreatic cell line

**Fig. 1:**
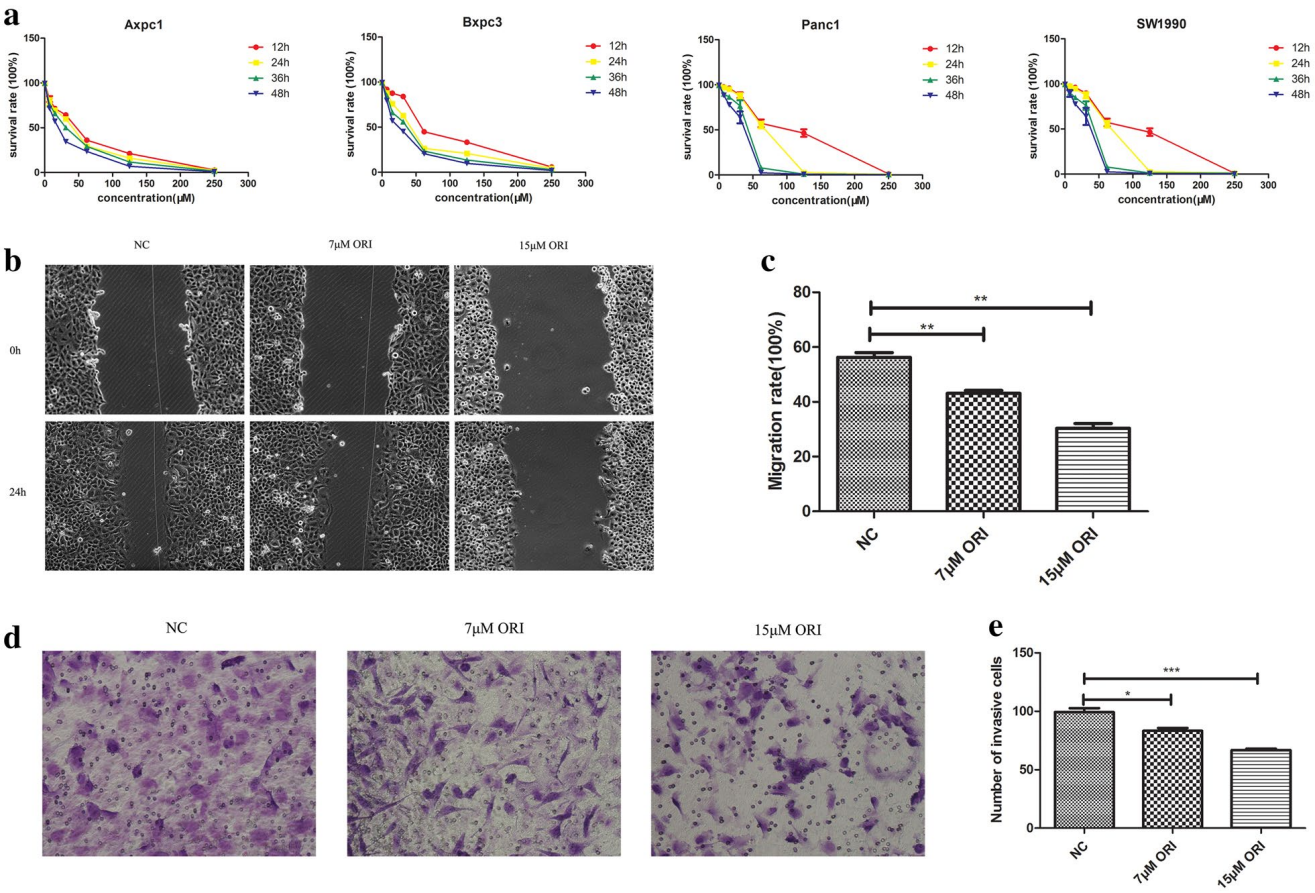
ORI inhibits SW1990 cell migration. **a** Cell viability was determined by using a CCK-8 assay. **b**–**e** Cell migration was determined by using wound healing and transwell assay. The migrative ability of ORI group (7 and 15 μM) was compared with that in the control group. *P < 0.05, **P < 0.01, ***P < 0.001 vs. control group

After treatment with different concentrations (7, 15, 31, 62, 125 or 250 μM) of ORI for 12, 24, 36 and 48 h, the proliferation rates of four human pancreatic cancer cell lines (Axpc1, Bxpc3, Panc1, SW1990) were assessed via CCK-8 kit. As shown in Fig. 1a, ORI exhibited anti-proliferative effects on all four cell lines in a concentration-dependent manner, with SW1990 being the least sensitive to ORI. Therefore, we decided to use this cell line, which was derived from a spleen metastasis of a grade II pancreatic adenocarcinoma [13], to evaluate the role of ORI in pancreatic cancer metastasis and EMT.

IC50 of ORI in SW1990 of 24 h was 63.47 μM (Table 1). ORI at doses higher than 30 μM inhibited cell growth. ORI concentrations of 7 and 15 μM were used to treat the cells for 24 h to evaluate the role of ORI in pancreatic cancer metastasis. The effect of ORI on cell migration was evaluated via wound healing and transwell migration assays. As shown in Fig. 1c and e, the migrative ability of SW1990 was significantly decreased after ORI treatment, compared with that in the control. Treatment with the 15 μM ORI concentration resulted in 46 % reduction in cell migrative ability (Fig. 1c; P < 0.05).

**Table 1:** IC50 of ORI

IC50 (μM)	12 h	24 h	36 h	48 h
Axpc1	38.68	32.25	26.27	17.99
Bxpc3	65.41	37.51	29.30	21.66
Panc1	86.83	63.46	38.76	32.49
SW1990	87.25	63.47	38.95	32.57

### EMT of SW1990 is affected by ORI

**Fig. 2:**
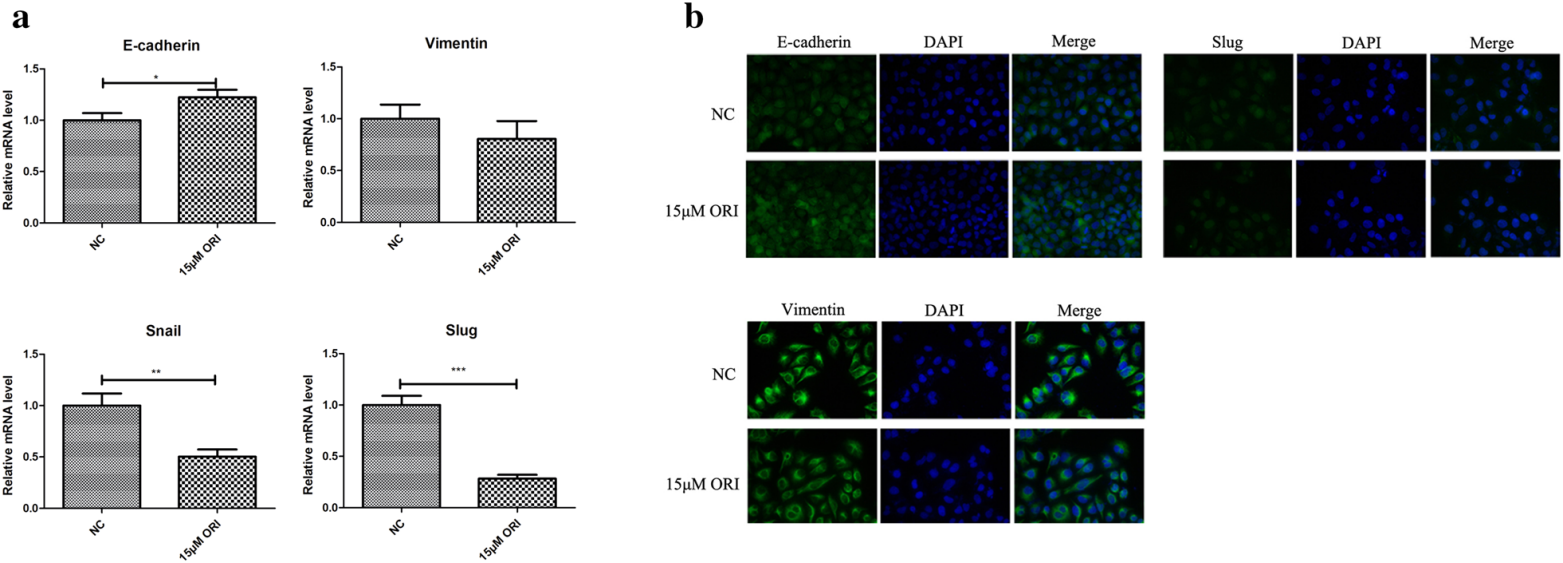
Impact of ORI on EMT of tumor cells. **a** Real-time PCR analysis of EMT markers were compared between control group and ORI group (15 μM). Data are presented as the mean ± SD, n = 3. **b** Immunofluorescence of EMT markers were compared between control group and ORI group (15 μM). *P < 0.05, **P < 0.01, ***P < 0.001 vs. control group

EMT is an early event in tumour migration. It is related to the structural changes in the intercellular junction, which is essential for migration [14]. We hypothesised whether the inhibition of tumour cell migration by ORI is associated with EMT. SW1990 were treated with 15 μM ORI for 24 h and evaluated for the expression of EMT markers by real-time PCR and immunofluorescent staining. Real-time PCR showed that the expression of mesenchymal markers, such as vimentin and transcription factors snail and slug, decreased, whereas the expression of epithelial-related genes E-cadherin was increased (Fig. 2a, b). However, immunofluorescent staining did not show significant changes of E-cadherin and slug.

### ORI inhibits β-catenin signalling in human pancreatic cancer cells

**Fig. 3:**
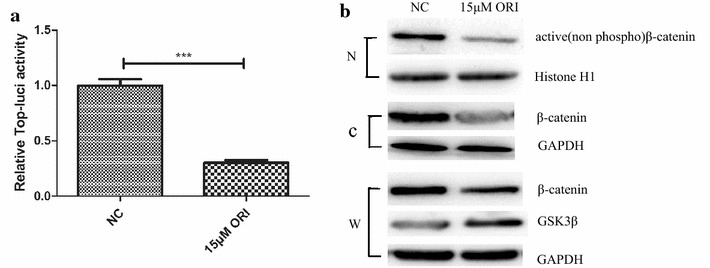
Functions of ORI relied on β-catenin and GSK3β. **a** Luciferase assays. **b** Representative western blots for active β-catenin, β-catenin(total) and GSK3β levels in control or ORI group. Histone H1 and GAPDH were used as internal control. (*N*, nucleus; *C*, cytoplasm; *W*, whole cell)

Given that stabilization and nucleus translocation of β-catenin are key events in transduction of the canonical Wnt/β-catenin signalling, which is highly involved in cancer metastasis, we conducted western blot analysis to explore whether oridonin can suppress β-catenin activity. The results indicated that ORI can decrease the β-catenin protein level not only in the nucleus, but also in the cytoplasm and the whole cell after the treatment with ORI for 24 h (Fig. 3b). Luciferase assays demonstrated that ORI dramatically suppressed the transcriptional activity of β-catenin (Fig. 3a). The stability of β-catenin in the cytoplasm is tightly regulated by the Axin/APC/GSK3β complex. Phosphorylation of β-catenin by GSK3β resulted in its degradation, which led to the inactivation of Wnt/β-catenin signalling [15–18].Thus, we investigated whether ORI could also affect the expression of GSK-3β, which is the negative regulator of Wnt/β-catenin signalling. As shown in Fig. 3b, the total expression level of GSK3β increased after ORI treatment. ORI may regulate Wnt/β-catenin signalling by enhancing the function of GSK3β in SW1990 cells.

### CHIR attenuates the inhibitory function of ORI

**Fig. 4:**
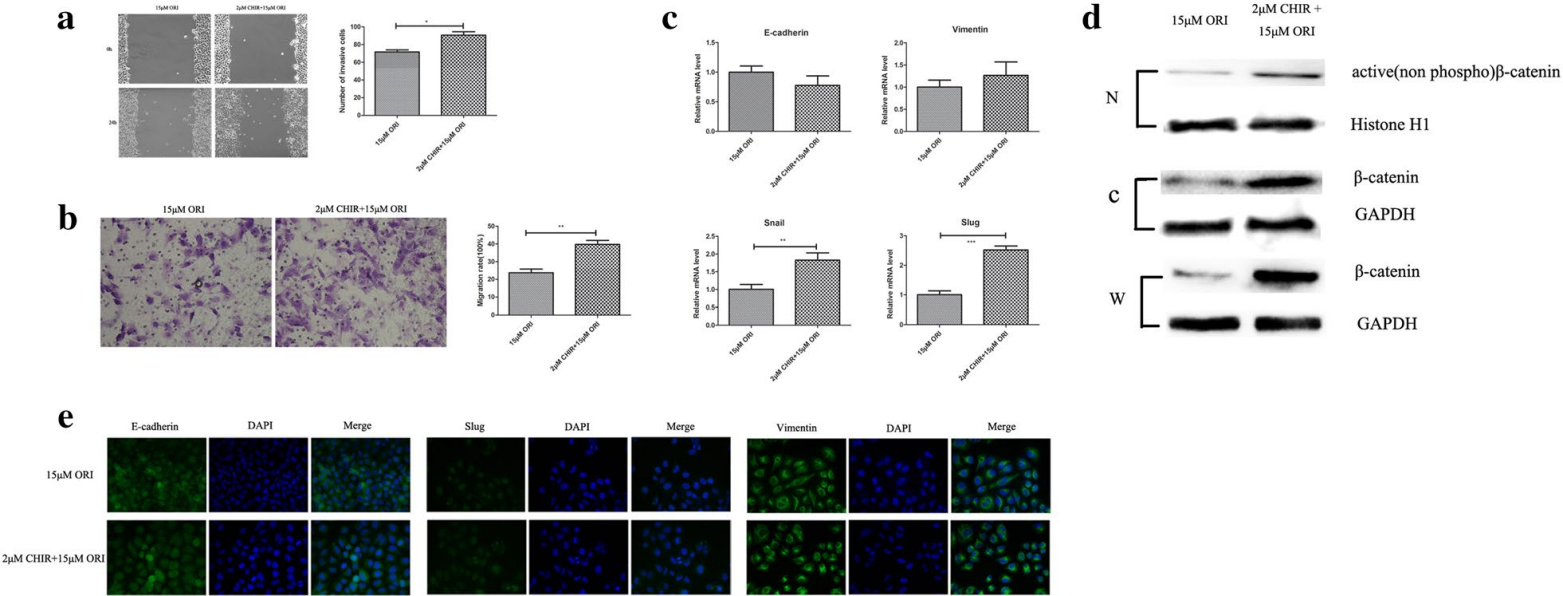
CHIR attenuated the inhibitory function of ORI. **a** and **b** Cell migration was determined by using wound healing and transwell assay. The migrative ability of CHIR group (2 μM CHIR + 15 μM ORI) was compared with that seen in the ORI group (15 μM). **c** Real-time PCR analysis of EMT markers were compared between CHIR group(2 μM CHIR + 15 μM ORI) and ORI group (15 μM). **d** Representative western blots for active β-catenin and β-catenin(total) levels in ORI or CHIR group. Histone H1 and GAPDH were used as internal control. (*N*, nucleus; *C*, cytoplasm; *W*, whole cell). **e** Immunofluorescence of EMT markers were compared between CHIR group (2 μM CHIR + 15 μM ORI) and ORI group (15 μM). Data are presented as the mean ± SD, n = 3. *P < 0.05, **P < 0.01, ***P < 0.001 vs. ORI group (15 μM)

To further validate the role of β-catenin and GSK3β in the migration inhibitory effect of ORI on SW1990 cells, SW1990 was tested with CHIR, a GSK-3-specific inhibitor. CHIR attenuated the inhibitory function of ORI. WB results indicated that CHIR can reverse the decrease of the β-catenin in the cytoplasm, nucleus and the whole cell (Fig. 4d). As shown in Fig. 4a and b, after simulation with CHIR, the inhibited migration of SW1990 in the ORI group (15 μM) was markedly improved. Furthermore, the expression of E-cadherin decreased, whereas, the expression of vimentin, snail and slug increased after CHIR treatment (Fig. 4c, e, P < 0.05). These results suggest that down-regulation of β-catenin plays a critical role in the function of ORI in SW1990 cells.

### ORI inhibits tumour metastasis in vivo

**Fig. 5:**
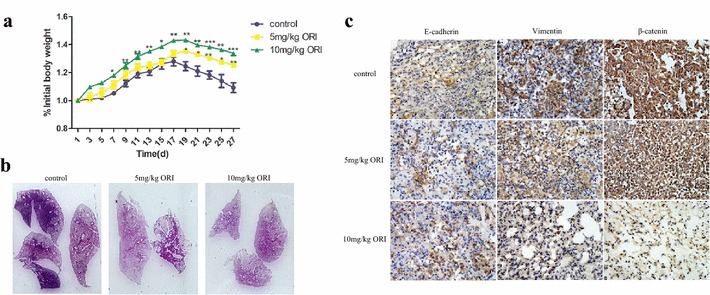
ORI inhibited tumor metastasis in vivo. **a** The change of body weight after injection of SW1990 and ORI. *P < 0.05, **P < 0.01, ***P < 0.001 vs control group. **b** Photographs of H&E staining of lung tissues from control or treatment (ORI) group were shown. **c** IHC images of lungs stained with E-cadherin, vimentin and β-catenin

We injected 1 × 10^6^ SW1990 cells into nude mice to test whether ORI affects tumour metastasis. As shown in Fig. 5a, ORI treatment significantly decreased the body weight loss compared with the control. In addition, on the fourth week, two of the five mice in the control group died. By contrast, no death was recorded in the ORI group (5 and 10 mg/kg). Furthermore, lung metastasis was significantly inhibited compared with the control group, even though no metastatic nodules could be observed by the naked eye. A representative lung from each group was shown in Fig. 5b. IHC analysis of tumour sections stained with anti-E-cadherin, anti-vimentin and anti-β-catenin antibodies revealed that ORI inhibited EMT as well as Wnt/β-catenin pathway in vivo (Fig. 5c).

## Discussion

ORI is a tetracycline diterpenoid compound extracted from the traditional Chinese medicinal plant *Rabdosia rubescens*. It exerts immune protective effects in severe inflammatory diseases. ORI has shown remarkable anti-proliferative and pro-apoptotic effects against leukemia and some types of solid tumours [19–23]. It also inhibits migration in various cancer cells [24–26]. ORI has been reported to significantly inhibit lung tumour metastasis through anti-angiogenesis by blocking Notch signalling; it also inhibits tumour invasion and metastasis in vitro possibly by decreasing the expression of MMPs and regulating the Integrin β1/FAK pathway in human breast cancer MDA-MB-231 cells. However, the effect of ORI on the migration of pancreatic cancer cells remains unclear. We investigated these mechanisms of ORI on pancreatic cancer cells, and ORI can inhibit pancreatic cancer cell SW1990 migration and EMT by down-regulating Wnt/β-catenin signal transduction.

EMT is defined by epithelial cells taking on a mesenchymal phenotype, characterised by loss of apical-basal polarity, increased cellular motility and reorganization of cytoskeleton [27]. There are three major types of EMT: type 1 refers to embryogenesis; type 2 refers to wound healing; and type 3 refers to cancer and metastases [28]. During the process of type 3 EMT, epithelial cells lose contact with their neighbours and gain mesenchymal properties, which enable them to break through the basement membrane for their metastatic dissemination. One of the hallmarks of EMT is the functional loss of E-cadherin (encoded by CDH1), which is thought to be a metastasis suppressor during tumour progression [29]. Vimentin and the transcription factors, snail and slug, are prominent inducers of EMT and strongly repress E-cadherin expression [30, 31]. Highly invasive breast cancer cells have been studied and selected, and these cells displayed EMT characteristics and dramatically enhanced invasive abilities with decreased levels of E-cadherin and increased vimentin, fibronectin, Twist and AKT2 [32]. Metastasis may be largely dependent on the ability of cancer cells to acquire EMT characteristics [33, 34]. Researchers found that paeoniflorin blocked the migration and invasion of breast cancer cells by repressing EMT under hypoxic conditions [35]. Cui speculated that asparaginyl endopeptidase could promote the invasion and metastasis of gastric cancer via EMT through AKT and MAPK signalling pathways [36]. Our results showed that the expression of mesenchymal molecular markers (vimentin, snail and slug) were decreased by ORI, whereas the expression of E-cadherin increased, indicating that ORI affects the EMT process in pancreatic cancer cells.

Wnt/β-catenin signalling pathway plays a critical role in various cancers [37, 38]. In the canonical Wnt/β-catenin signalling, Wnt ligands bind to the dual receptor complex comprised of frizzled and low-density lipoprotein receptor-related protein 5/6 (LRP5/6). This leads to inactivation of the β-catenin destruction complex and Axin/APC/GSK-3β; the critical mediator β-catenin is relieved from its constitutive proteosomal degradation. β-catenin subsequently accumulates in the cytoplasm and translocates into the nucleus, where it associates with transcription factors to regulate the downstream target genes [39]. Increased cytoplasmic and/or nuclear accumulation of β-catenin protein is a common feature in human pancreatic cancers and it is involved in its pathogenesis [40]. The compounds from Chinese herbal medicine targeting Wnt/β-catenin signalling can be considered as suitable candidates for pancreatic cancer treatment. Given that ORI could activate GSK3β activity and up-regulate the expression of DKK1 in human osteosarcoma [41], the anti–migratory effect of ORI on pancreatic cancer cells might result from targeting Wnt/β–catenin signalling. Our data showed that ORI can reduce the protein levels of active β-catenin and its transcriptional activity in the nucleus. Moreover, inhibiting phosphorylation of GSK3β could attenuate the function of ORI on migration and EMT in SW1990. In addition, ORI-treated tumour xenografts express much lower levels of β-catenin compared with control tumours. It is then very likely that the effect of ORI on pancreatic cancer metastasis is due to its suppressive effect on β-catenin signalling.

## Conclusions

Our data suggest that ORI can be effective for human pancreatic cancer. The anticancer effect of ORI in SW1990 cells may result from the suppression of EMT by increasing GSK-3β activity and inactivating Wnt/β-catenin signal transduction. Future studies should be directed to the identification of more ORI target proteins, which are essential to elucidate the molecular mechanisms underlying the anti-cancer capacity of ORI.

## Abbreviations

ORI: oridonin
EMT: epithelial-to-mesenchymal transition
GSK3β: glycogen synthase kinase 3β
DKK-1: dickkopf-1
